# SAPHIRE: Stress and Pulmonary Hypertension in Rheumatoid Evaluation—A Prevalence Study

**DOI:** 10.1155/2016/4564531

**Published:** 2016-04-20

**Authors:** G. E. M. Reeves, N. Collins, P. Hayes, J. Knapp, M. Squance, H. Tran, B. Bastian

**Affiliations:** ^1^John Hunter Hospital, University of Newcastle, Callaghan, NSW 2308, Australia; ^2^Department of Cardiology, John Hunter Hospital, New Lambton Heights, NSW 2305, Australia; ^3^Autoimmune Resource and Research Centre, New Lambton Heights, NSW 2305, Australia; ^4^Pathology North, New Lambton Heights, NSW 2305, Australia

## Abstract

Pulmonary artery hypertension (PAH) is a disorder of elevated resistance in the pulmonary arterial vessels, reflected by elevation of measured pulmonary artery pressure (PAP), and presenting with breathlessness and, if untreated, progressing to right heart failure and death. The heightened prevalence of PAH in populations with underlying systemic autoimmune conditions, particularly scleroderma and its variants, is well recognised, consistent with the proposed autoimmune contribution to PAH pathogenesis, along with disordered thrombotic, inflammatory, and mitogenic factors. Rheumatoid arthritis (RA) is one of a group of systemic autoimmune conditions featuring inflammatory symmetrical erosive polyarthropathy as its hallmark. This study explored the prevalence of PAH in a population of unselected individuals with RA, using exercise echocardiography (EchoCG). The high prevalence of EchoCG-derived elevation of PAP (EDEPP) in this population (14%) suggests that, like other autoimmune conditions, RA may be a risk factor for PAH. Patients with RA may therefore represent another population for whom PAH screening with noninvasive tools such as EchoCG may be justified.

## 1. Introduction

The heightened prevalence of PAH in individuals with scleroderma, combined with the arrival of specific therapies which improve PAH prognosis, has led to widespread screening for PAH in this patient subgroup in accordance with published guidelines [1]. Subsequently, patients with other autoimmune conditions (e.g., SLE [2, 3] and Sjögren's syndrome [4]) have also been found to display increased prevalence of PAH, and similar screening recommendations have been made for these groups [5]. The proposal that PAH is a form of autoimmune disease has been increasingly advocated [6], based upon these associations as well as shared clinical, serological, and other features [7].

Rheumatoid arthritis (RA) is an autoimmune condition which affects the rheumatological system but may involve the vasculature, eyes, lungs, and other organs. The shared pathogenesis between rheumatoid and other autoimmune conditions has raised the question of whether patients with RA may also display a heightened prevalence of PAH [8], although some large studies contradict this suggestion [9, 10], and there is not presently conclusive evidence addressing this issue. Previous studies have found that the inclusion of stress associated peak systolic pulmonary artery pressure (PASP) elevation may increase sensitivity for detection of PAH in at-risk populations, such as individuals with scleroderma [11], although consensus recommendations still advocate caution in the use of such measures due to lack of normative data [12].

The Stress Associated Pulmonary Hypertension in Rheumatoid Evaluation (SAPHIRE) study was conducted to explore the prevalence of pulmonary hypertension in individuals with rheumatoid arthritis (RA) using stress echocardiography (SE) as a screening tool.

## 2. Patients and Methods

### 2.1. Patient Selection and Study Design

The Autoimmune Resource and Research Centre (ARRC) was established in 1990 to provide education and support for individuals with systemic and organ-specific autoimmune syndromes. Using the ARRC database and individuals seen by the Rheumatology Clinic of the Royal Newcastle Centre, 177 subjects with American College of Rheumatology (ACR) criteria confirmed rheumatoid arthritis (RA) were randomly invited to participate in the study. Recruited patients were evaluated via stress echocardiography. Inclusion criteria included age ≥ 18 years, weight ≥ 40 kg, and ability to undergo informed consent; individuals with total lung capacity (TLC) < 60% of predicted volume were excluded, as were patients with previous pulmonary thromboembolism. Predetermined withdrawal criteria included worsening rheumatoid inflammation precluding further participation or leading to death or change in inclusion criteria status (e.g., pregnancy). The study proceeded in accordance with the 1983 revision of the 1975 Helsinki Declaration and in keeping with guidelines for good clinical practice. The trial protocol was reviewed by the Hunter Area Research Ethics Committee.

### 2.2. Variables and Measurements

Patients were asked to complete the World Health Organisation (WHO) functional class assessment, standardised six-minute walk test (6MWT), and formal pulmonary function testing (PFT) including carbon dioxide diffusing capacity (DLCO). WHO functional assessments are classified as follows: Class I (unlimited during activity); Class II (some breathlessness with extreme exertion); Class III (breathlessness interfering with usual activities of daily living); and Class IV (breathlessness at rest).

**Figure 1:**
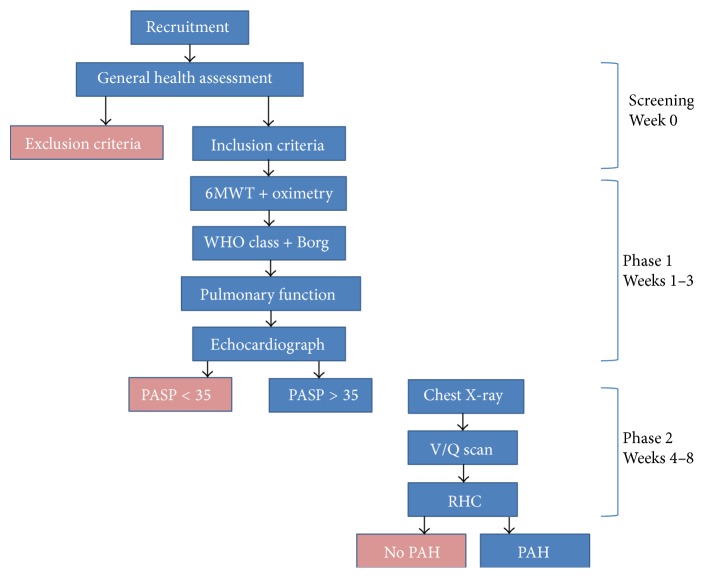
SAPHIRE study design.

Patients were assessed for raised PASP (echocardiographically derived elevation of pulmonary pressure, EDEPP) at rest and following exercise using SE; if unable to exercise, they underwent resting echocardiography only. Based upon literature review [11], it was estimated that 10% of recruited participants could require supplementary assessment (Phase 2). Chest X-rays and ventilation-perfusion (V/Q) scanning were performed on all individuals with PASP > 35 mmHg, to address and exclude possible pulmonary parenchymal and microvascular thrombotic problems, respectively (see Figure 1).

### 2.3. Laboratory Studies

Analysis of biochemical markers, such as B type natriuretic peptide and troponin I, was not included in the study design. C-reactive protein (CRP) plasma concentrations were measured with an ultrasensitive latex immunoassay (Architect analyser, Abbott) and antibodies to cyclic citrullinated peptide (CCP) were measured by enzyme immunoassay (EIA) (Immunoscan RA CCP3, Euro-Diagnostica, Arnhem, the Netherlands) according to the manufacturer's details.

### 2.4. Doppler Echocardiography with Exercise

Patients undertook exercise stress testing using a modified Naughton protocol with standard 12 lead electrocardiogram (ECG) and noninvasive blood pressure monitoring. All echocardiograph studies were performed by a single sonographer and interpreted by a single experienced cardiologist, blinded to full clinical case details. Echocardiograph measures of left ventricular function were also performed before and after exercise. Continuous wave Doppler interrogation of the tricuspid regurgitant (TR) jet was used to estimate peak systolic pulmonary artery pressure (PASP), which is considered to be the most accurate echocardiographic method. TR jet interrogation allows estimation of the peak right ventricle to right atrium gradient using the modified Bernoulli equation (4*V* ^2^ + right atrial (RA) pressure, with RA pressure assumed as 10 mmHg in keeping with previously published data when employing exercise stress echocardiography in patients with autoimmune disease [2]). The assumed RA pressure eliminates the possibility of error in the clinical examination of the jugular venous pressure or assessment of inferior vena cava size and its response to respiration. The possibility of WHO Group 2 PH (postcapillary PH) was excluded by confirmation of normal LV systolic and diastolic function on standardised echocardiographic reports. These values were assessed before (PASPr) and after (PASPe) exercise, with rise in exercise echo pressure (REEP) calculated as (PASPe – PASPr). TR jet measurement estimates PASP in up to 90% of patients [5], with significant correlation with the results of right heart catheterisation (RHC) [13]. PAH has been defined previously as an elevated resting mean pulmonary artery pressure (PAP) > 25 mmHg at rest or by an elevation in mean PAP with exercise (>30 mmHg with exercise). When using stress echocardiography, PASP at rest > 35 mmHg (resting, PASPr) and following exercise > 40 mmHg (postexercise, PASPe) was considered abnormal. Participants found to have a likely diagnosis of PAH (as reflected by elevated EchoCG-defined PASP (EDEPP) using these criteria) were referred to the interdisciplinary PAH clinic for assessment and appropriate treatment and management.

### 2.5. Right Heart Catheterisation

RHC was performed under aseptic conditions in the cardiac catheterisation laboratory after informed consent was obtained. Following femoral venous access, a Swan Ganz catheter was advanced into the pulmonary artery and pressure measurements were obtained, including pulmonary artery capillary wedge pressure. Cardiac output assessment, required for assessment of pulmonary vascular resistance, was obtained using both Fick and thermodilution methods.

### 2.6. Features of Patients with PAH

According to the protocol, RHC assessment was offered for all patients displaying EDEPP. However, given the invasiveness of the procedure and the lack of conclusive benefit from early intervention [14], many elected not to proceed, with any subsequent decision to proceed to right heart catheterisation left to the discretion of the patient and their primary carer.

### 2.7. Statistical Analysis

To determine prevalence rates with a precision of 7%, a sample size of 71 patients was required, assuming a 10% prevalence rate for EDEPP in rheumatoid patients in our sample (95% confidence interval for prevalence rate of 4% to 19%) [15].

Primary endpoints were prevalence of EDEPP in patients with rheumatoid arthritis, and, where possible, confirmation of PAH. Two outcomes were analysed: (i) PAH prevalence as determined by PASPr > 35 (EchoCG) and/or mean pulmonary artery pressure (MPAP) > 25 mmHg at rest (RHC) and (ii) PAH prevalence as determined by PASPe > 40 (EchoCG) and/or mean PAP > 30 with exercise (RHC).

Clinical and measured variables are summarized as means ± SD (or median with range where appropriate) for continuous data and as frequencies (with percentages) for categorical data. Differences between these summary statistics are analysed using the Student *t* or Mann-Whitney-Wilcoxon tests where appropriate for continuous variables and the chi-square test (or exact test where appropriate) for categorical variables.

A probability value of *p* < 0.05 (two-sided) is considered significant. Analyses were performed using Stata version 11 (Stata Corporation, College Station, Texas).

## 3. Results

### 3.1. Population Features

177 patients were invited to participate in the SAPHIRE study, of whom 100 patients responded and were accepted. A final number of 80 were included in the study; 7 people withdrew, and 13 were excluded based upon predefined exclusion criteria. All patients declining involvement in the study expressed concern surrounding potential performance of invasive RHC studies in the context of subjectively good functional capacity and lack of clear therapeutic gain.

80 patients were studied, displaying a mean age of 60 (80% female), dominantly falling into WHO Class II functional class. Overall mean values for key variables are listed in Table 1. Only two patients in this study displayed Raynaud's phenomenon.

The diagnosis of PAH was based predominantly on echocardiographic findings, reflecting patient reluctance to undergo invasive testing with RHC (Table 2).

**Figure 2:**
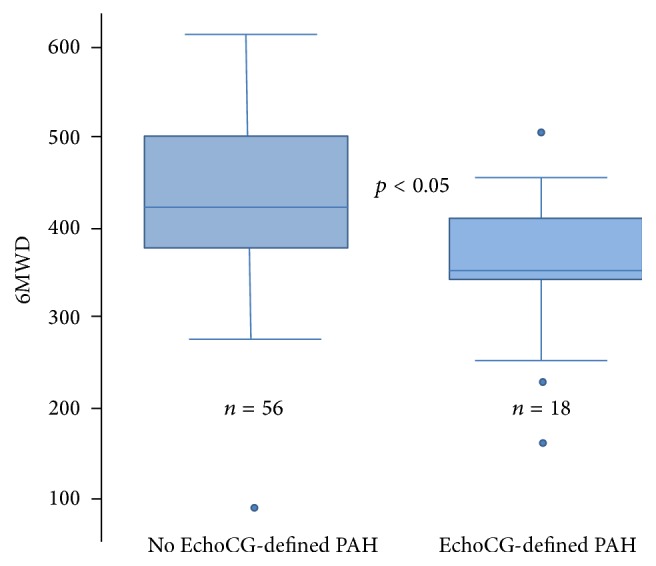
Six-minute walk distance by EchoCG-derived PAH.

**Figure 3:**
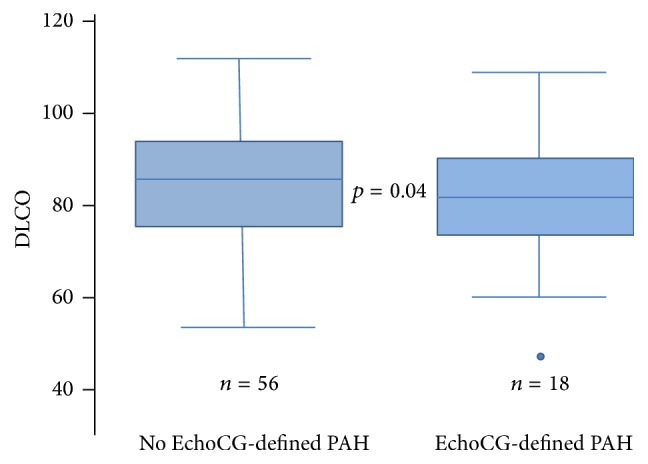
DLCO by EchoCG-derived PAH.

In patients diagnosed with PAH, as expected, PASP (as determined by EchoCG) was significantly higher compared to those without PAH. Resting and postexercise PASP values were approximately 15 mmHg greater in the PAH groups versus those without this condition (PASPr (mmHg): 40.8 (with PAH) versus 25.5 (without PAH) (*p* < 0.05); PASPe (mmHg): 42.5 (with PAH) versus 28.2 (without PAH) (*p* < 0.05)). There was no significant difference between subgroups for the exercise-induced rise in PASP (REEP), reflecting resting PA pressure as an important discerning echocardiographic parameter. There were no significant differences in the serological markers CRP and CCP across patient groups with and without PAH. Significant reductions in DLCO (78.8 versus 85.3% predicted, *p* = 0.04) and 6MWD (356.8 versus 441.3 m, *p* < 0.05) were also noted in patients with EDEPP as compared to those without. These significant relationships are represented graphically in Figures 2 and 3.

Using the cutoffs for PASP elevation defined in this study, it was determined that the prevalence of PAH in patients with RA is 14% (using a resting PASP cutoff of > 35 mmHg), increasing to 21% when elevations in SE derived pulmonary pressures are considered. Of the small number of RA patients who agreed to proceed to right heart catheterisation studies, 2 out of 6 (33%) were diagnosed with PAH (Table 3).

## 4. Discussion

The prevalence of pulmonary hypertension in RA is not well reported, with some authors stating it is rare [9, 10, 16] and others finding prevalence rates as high as 27% [17]. This study demonstrates that there is a significant prevalence of EchoCG-defined PH in patients with rheumatoid arthritis, with 14% of RA patients displaying elevation of resting PASP > 35 mmHg.

PAH is often silent, with the development of clinical symptoms often signalling the presence of advanced disease, associated with poor survival. With available therapeutic options (including phosphodiesterase inhibitors, prostacyclin analogues, and dual-receptor or single-receptor (ET-A) endothelin blockade) and given the irreversibility of advanced pathology associated with PAH, an impetus is provided for earlier detection of PAH in at-risk populations, with exercise proposed as a useful stress tool, albeit a method yet to possess clear normal cutoff ranges.

For screening to be justified, a condition must be common enough in the at-risk population to warrant the inconvenience, false positives, medicalisation, and potential costs of the screening strategy. Pulmonary hypertension screening has been associated with improved survival displayed in the subgroup of scleroderma patients in whom earlier PAH detection was secured [18]. The prevalence of EDEPP in our study is similar to that seen in other conditions such as scleroderma, where routine population screening is established.

Screening tools such as EchoCG, whilst imperfect in terms of sensitivity and specificity against the “gold standard” of RHC, are still useful with high pretest probability, such as in screening high risk groups including scleroderma, systemic lupus erythematosus, and now possibly rheumatoid arthritis. Given the inherent limitations of any single parameter in determining the likelihood of pulmonary hypertension in a given individual, expert consensus is increasingly moving towards multiparametric approaches incorporating more than one predictor variable to refine determination of pulmonary hypertension risk. In our study, the prevalence of PAH was increased with the incorporation of SE, although the clinical relevance and specificity of these findings require further study. The use of assumed right atrial pressure may potentially overestimate the true prevalence of pulmonary hypertension in this population. This overly sensitive result may be appropriate in terms of screening to avoid false negative results, given the deleterious effects of late diagnosis. The use of DLCO represents an additional modality with previously published data demonstrating that the prevalence of PAH was enriched approximately fivefold when testing incorporated a DLCO < 55% [19, 20].

It is acknowledged that the diagnostic gold standard test for defining PAH is the right heart catheter and that the key parameters in this condition include pulmonary vascular resistance and right heart function, as well as exclusion of coexisting left heart disease by measurement of pulmonary capillary wedge pressure. Whilst RHC determines mean PAP (mPAP), the EchoCG-derived value estimates only peak systolic PAP, as calculated from tricuspid regurgitant jet velocity. Lack of systematic right heart catheterisation is a limitation of this study and is reflected in previous reports which have highlighted the imperfect correlation between PASP as derived from EchoCG and mean PAP measured during RHC. However, echocardiographically based screening as an initial approach in these patient populations is advocated because (i) RHC is impractical as a tool for identifying PAH tendencies in at-risk populations, where noninvasive strategies are required; (ii) although linear correlation between PASP (Echo-derived) and mPAP (from RHC) is imperfect, the agreement between methods is acceptable when a higher threshold for EchoCG-derived PASP is employed [21]; and (iii) EchoCG-based tools are more amenable to the incorporation of exercise components for unmasking latent PAH tendencies. The reliability of EchoCG for defining a subgroup of our patients with functional compromise in the context of PAH is supported by the clear demonstration of significant reductions of DLCO and 6MWD in patient groups displaying EDEPP. The invasiveness of RHC and the lack of definitive data for improved outcomes with early therapy create difficulties in this and other similar prospective screening studies.

Whilst the proposed study protocol was designed to clarify abnormal EchoCG-derived PAP measurements, the decision to proceed to RHC was contingent upon patient and clinician agreement; reluctance was expressed in the study group surrounding potential performance of invasive RHC studies in the context of subjectively good functional capacity, potential RHC risks, and lack of clear therapeutic gain (particularly as risk-benefit gain with early treatment of PAH (WHO functional Class I) is still contentious [20]). Using adjusted cutoffs, the diagnostic performance of EchoCG-derived PAP in detecting PAH compares favourably with RHC-based diagnosis, as reflected by good correlation between the two methods—for example, Spearman's rho correlation value of 0.92 (*p* < 0.001) was confirmed in a recent study [22]. Of note, a significant proportion of patients were in WHO functional Class II or above, potentially reflecting a volunteer bias, although this mild degree of dyspnoea may also reflect deconditioning, a frequent problem in RA patients [23].

It has been proposed that some of the pulmonary vascular pathology underlying PAH may reflect a similar phenomenon to the peripheral vascular hyperreactivity seen in Raynaud's phenomenon, possibly reflecting similar vasoconstrictor-mediated pathophysiology. Due to a lack of patients affected by Raynaud's phenomenon in our patient cohort (only two patients with Raynaud's phenomenon), this question could not be addressed in this study.

The clinical significance of exercise-induced EDEPP is controversial, with consensus guidelines having suggested that exercise EchoCG not be employed routinely in the absence of normative values [13]. It is noted that the ability to increase cardiac output may produce elevation in the PAP that reflects a normal response of the pulmonary vasculature to exercise; the presence of comorbidity in these patients is likely to result in diminished exercise reserve, with increases in PAP more likely to reflect abnormalities of the pulmonary circulation rather than marked increase in cardiac output. Previous work by this group and others suggests that the ability to physiologically unmask latent pulmonary hypertensive tendencies with EchoCG provides valuable additional information in assessing possible contribution of PH to exertional dyspnoea symptoms [2], and there is evidence that such tendencies may antedate progression to resting PAH. However, the potential role of such functional measures is still argued, with some authorities suggesting that exercise EchoCG can be helpful in specified clinical situations [24]. In this study, 21% of the patients displayed elevation of PASP with exercise (compared with 14% on resting EchoCG alone). Although our study showed that rise in PASP with exercise (REEP) provided no additional diagnostic information, the prevalence and clinical significance of this functional, subclinical form of PAH in RA warrant further prospective study.

The shared clinical, serological, and epidemiological features seen in both PAH and other autoimmune conditions led us to explore the possible roles of autoimmunity and inflammation in our disease subset with abnormal PASP. Whilst the patients with and without PAH displayed no significant difference in CRP or CCP values, the heightened prevalence of EDEPP and PAH in our cohort of RA patients fits well within an autoimmune paradigm for PAH pathogenesis.

## 5. Conclusions

This study screened a population of patients with RA, irrespective of the presence of breathlessness at baseline, with rest and stress echocardiography to assess the prevalence of PAH in this group. We demonstrated that there is a significant prevalence of EchoCG-defined PH in patients with RA, with 14% of RA patients displaying elevation of resting PASP > 35 mmHg.

The demonstration of an increased prevalence of PAH in this population of RA patients suggests that RA may be considered along with other systemic autoimmune conditions (currently including scleroderma, lupus, and sarcoidosis) for which periodic clinical and further assessment for PAH needs to be regularly considered. Whilst this proposed 14% prevalence of PAH is inferred largely from noninvasive data using an established threshold for pulmonary hypertension based on echocardiography, it warrants further replication and, ideally, exploration with routine RHC to confirm the presence of PAH.

## Figures and Tables

**Table 1:** Patient characteristics (*n* = 80).

Age (yrs)	60.0 ± 10.1
Gender (male : female)	32 : 48
WHO Class II	77 (96.2%)
WHO Class III	3 (3.8%)
BMI (kg·m^−2^)	30.0 ± 6.2
CRP (mg·L^−1^)	29.8 ± 50.0
CCP (U·L^−1^)	122.7 ± 126.7
PASPr (resting, mmHg)	29.6 ± 12.0
PASPe (exercise, mmHg)	33.0 ± 9.7
REEP (rise in exercise echo pressure)	4.9 ± 11.1
DLCO (% predicted)	83.8 ± 13.7
6MWD (m)	415.7 ± 107.5

**Table 2:** Patient characteristics by PAH status (*n* = 80).

	PAH determined by RHC (*n* = 6)			PAH determined by EchoCG (*n* = 74)		
	No PAH (*n* = 4 [67%])	PAH (*n* = 2 [33%])	*p* value	No PAH (*n* = 56 [76%])	PAH (*n* = 18 [24%])	*p* value
Age (yrs)	62.3 ± 9.3	58.0 ± 12.7	0.66	60.0 ± 9.8	60.6 ± 11.3	0.82
WHO Class II	4	2	—	56	15	—
WHO Class III	0	0	—	0	3	—
BMI (kg·m^−2^)	32.5 ± 5.2	35.7 ± 7.3	0.06	29.0 ± 5.6	31.1 ± 5.7	0.16
CRP (mg·L^−1^)	20.2 ± 20.0	4.1 ± 0	—	32.7 ± 57.3	23.5 ± 21.0	0.55
CCP (U·L^−1^)	223 ± 158.1	312 ± 0	—	111.8 ± 123.4	110.8 ± 115.9	0.98
PASPr (resting) (mmHg)	31.8 ± 4.5	37.5 ± 14.8	0.47	25.5 ± 3.3	40.8 ± 20.4	<0.05
PASPe (exercise) (mmHg)	48.8 ± 15.7	51.5 ± 9.2	0.84	28.2 ± 5.0	42.5 ± 5.8	<0.05
REEP (rise with exercise)	17 ± 12.2	14 ± 5.7	0.77	3.1 ± 4.5	6.3 ± 21.3	0.31
DLCO (% predicted)	80.8 ± 14.2	92 ± 14.1	0.41	85.3 ± 13.3	78.8 ± 14.2	0.04
6MWD (m)	382.8 ± 19.2	290 ± 12.7	0.18	441.3 ± 105.8	356.8 ± 92.7	<0.05

**Table 3:** PAH prevalence in RA (*n* = 80).

Elevated mean PAP (RHC)	2/6 (33%)
Elevated resting PASP (EchoCG)	11/80 (14%)
Elevated exercise PASP (EchoCG)	17/80 (21%)

## Abbreviations

SAPHIRE: Stress and pulmonary hypertension in rheumatoid evaluation
PAH: Pulmonary artery hypertension
PAP: Pulmonary artery pressure
RA: Rheumatoid arthritis
EchoCG: Echocardiography
SE: Stress echocardiography
EDEPP: Elevation of echocardiographically derived PAP
PASP: Pulmonary artery systolic pressure (echocardiographically derived)
PASPr: Pulmonary artery systolic pressure (resting)
PASPe: Pulmonary artery systolic pressure (exercise)
MPAP: Mean pulmonary artery pressure (RHC-derived)
REEP: Rise with exercise in echo PASP.

## References

[1] Galie N., Hoeper M. M., Humbert M. (2009). Guidelines for the diagnosis and treatment of pulmonary hypertension. The Task Force for the Diagnosis and Treatment of Pulmonary Hypertension of the European Society of Cardiology (ESC) and the European Respiratory Society (ERS), endorsed by the International Society of Heart and Lung Transplantation (ISHLT). *European Respiratory Journal*.

[2] Collins N., Bastian B., Quiqueree L., Jones C., Morgan R., Reeves G. (2006). Abnormal pulmonary vascular responses in patients registered with a systemic autoimmunity database: pulmonary Hypertension Assessment and Screening Evaluation using stress echocardiography (PHASE-I). *European Journal of Echocardiography*.

[3] Prabu A., Patel K., Yee C.-S. (2009). Prevalence and risk factors for pulmonary arterial hypertension in patients with lupus. *Rheumatology*.

[4] Kobak S., Kalkan S., Kirilmaz B., Orman M., Ercan E. (2014). Pulmonary arterial hypertension in patients with primary sjögren's syndrome. *Autoimmune Diseases*.

[5] McGoon M., Gutterman D., Steen V. (2004). Screening, early detection, and diagnosis of pulmonary arterial hypertension: ACCP evidence-based clinical practice guidelines. *Chest*.

[6] Mouthon L., Guillevin L., Humbert M. (2005). Pulmonary arterial hypertension: an autoimmune disease?. *European Respiratory Journal*.

[7] Nicolls M. R., Taraseviciene-Stewart L., Rai P. R., Badesch D. B., Voelkel N. F. (2005). Autoimmunity and pulmonary hypertension: a perspective. *European Respiratory Journal*.

[8] Dawson J. K., Goodson N. G., Graham D. R., Lynch M. P. (2000). Raised pulmonary artery pressures measured with Doppler echocardiography in rheumatoid arthritis patients. *Rheumatology*.

[9] Condliffe R., Kiely D. G., Peacock A. J. (2009). Connective tissue disease-associated pulmonary arterial hypertension in the modern treatment era. *American Journal of Respiratory and Critical Care Medicine*.

[10] Chung L., Farber H. W., Benza R. (2014). Unique predictors of mortality in patients with pulmonary arterial hypertension associated with systemic sclerosis in the REVEAL registry. *Chest*.

[11] Codullo V., Caporali R., Cuomo G. (2013). Stress doppler echocardiography in systemic sclerosis: evidence for a role in the prediction of pulmonary hypertension. *Arthritis and Rheumatism*.

[12] Kovacs G., Berghold A., Scheidl S., Olschewski H. (2009). Pulmonary arterial pressure during rest and exercise in healthy subjects: a systematic review. *European Respiratory Journal*.

[13] Homma A., Anzueto A., Peters J. I. (2001). Pulmonary artery systolic pressures estimated by echocardiogram vs cardiac catheterization in patients awaiting lung transplantation. *The Journal of Heart and Lung Transplantation*.

[14] Galiè N., Hoeper M. M., Humbert M. (2009). Guidelines for the diagnosis and treatment of pulmonary hypertension: the Task Force for the Diagnosis and Treatment of Pulmonary Hypertension of the European Society of Cardiology (ESC) and the European Respiratory Society (ERS), endorsed by the International Society of Heart and Lung Transplantation (ISHLT). *European Heart Journal*.

[15] Naing L., Winn T., Rusli B. N. (2006). Practical issues in calculating the sample size for prevalence studies. *Archives of Orofacial Sciences*.

[16] Young I. D., Ford S. E., Ford P. M. (1989). The association of pulmonary hypertension with rheumatoid arthritis. *Journal of Rheumatology*.

[17] Udayakumar N., Venkatesan S., Rajendiran C. (2008). Pulmonary hypertension in rheumatoid arthritis—relation with the duration of the disease. *International Journal of Cardiology*.

[18] Humbert M., Coghlan J. G., Khanna D. (2012). Early detection and management of pulmonary arterial hypertension. *European Respiratory Review*.

[19] Mukerjee D., St George D., Knight C. (2004). Echocardiography and pulmonary function as screening tests for pulmonary arterial hypertension in systemic sclerosis. *Rheumatology*.

[20] Hachulla E., Gressin V., Guillevin L., Carpentier P., Diot E., Sibilia J. (2005). Early detection of pulmonary artery hypertension in systemic sclerosis: a French nationwide prospective multicentre study. *Arthritis and Rheumatism*.

[21] Farber H. W., Foreman A. J., Miller D. P., Mcgoon M. D. (2011). REVEAL registry: correlation of right heart catheterization and echocardiography in patients with pulmonary arterial hypertension. *Congestive Heart Failure*.

[22] Gopal D. M., Doldt B., Finch K., Simms R. W., Farber H. W., Gokce N. (2014). Relation of novel echocardiographic measures to invasive hemodynamic assessment in scleroderma-associated pulmonary arterial hypertension. *Arthritis Care and Research*.

[23] Ekdahl C., Broman G. (1992). Muscle strength, endurance, and aerobic capacity in rheumatoid arthritis: a comparative study with healthy subjects. *Annals of the Rheumatic Diseases*.

[24] D'Alto M., Ghio S., D'Andrea A. (2011). Inappropriate exercise-induced increase in pulmonary artery pressure in patients with systemic sclerosis. *Heart*.

